# Estradiol treatment induces both shared and unique gene regulation and networks in adipose cell types of gonadectomized obese XX and XY mice

**DOI:** 10.1186/s13293-026-00859-z

**Published:** 2026-02-23

**Authors:** Yutian Zhao, Ruoshui Liu, Jonathan P. Ng, Sophia Yu, In Sook Ahn, Graciel Diamante, Guanglin Zhang, Ariel Thorson, Kelsey P. Schaefers, John M. Stafford, Xia Yang

**Affiliations:** 1 Department of Integrative Biology and Physiology, University of California, Los Angeles, CA USA; 2 Molecular, Cellular, and Integrative Physiology Interdepartmental Ph.D. Program, University of California, Los Angeles, CA USA; 3 Bioinformatics Interdepartmental Ph.D. Program, University of California, Los Angeles, CA USA; 4 Department of Molecular, Cell and Developmental Biology, University of California, Los Angeles, CA USA; 5 Department of Medicine, Division of Endocrinology, Diabetes, and Metabolism, The Ohio State University, Wexner Medical Center, Columbus, OH USA; 6 Department of Molecular Physiology & Biophysics, Vanderbilt University, Nashville, TN USA; 7 Department of Molecular and Medical Pharmacology, University of California, Los Angeles, CA USA

**Keywords:** Estradiol, Estrogen therapy, Visceral adipose tissue, Adipose stem and progenitor cell, Macrophage, Single-cell RNA sequencing, Obesity

## Abstract

**Background:**

Obesity is driven by the pathological expansion and accumulation of adipose tissue and demonstrates sex differences. Estradiol (E2) is known to influence fat distribution and metabolism. However, a comprehensive understanding of the sex-specific effect of E2 on individual adipose cell types remains elusive.

**Methods:**

We measured adiposity and utilized single-cell RNA sequencing to dissect how E2 affects the molecular processes within gonadal adipose tissue from diet-induced obese, gonadectomized mice of both sexes (XX and XY) through differential gene expression, pathway enrichment, transcription factor enrichment, intracellular and intercellular network modeling, and human disease relevance analysis.

**Results:**

We found striking sex- and cell-type-specific responses to E2 treatment. Accompanying more significant fat reduction under diet-induced obesity in XX mice, adipose stem and progenitor cells (ASPCs) of XX mice exhibited a stronger transcriptomic shift in response to E2 than ASPCs in XY mice, with altered expression of genes related to stemness and lipid metabolism. E2 broadly suppressed extracellular matrix (ECM) genes in both sexes, with more pronounced downregulation of collagen, glycoprotein, and metalloproteinase-related genes in XY preadipocytes, and reduced proteoglycan genes in XX mice. Macrophages also demonstrated heightened sensitivity to E2, showing trends in decreased proportion of lipid-associated macrophages, increased perivascular-like macrophages, and downregulated inflammatory and metabolic pathways in both sexes as well as sex-specific changes in immune genes. Furthermore, we identified strengthened macrophage to ASPC communications in XX mice and differential enrichment patterns of sex-biased E2-altered genes with human metabolic diseases.

**Conclusions:**

Our findings provide a cell-resolution, sex-specific understanding of E2’s profound impact on gonadal adipose tissue remodeling to guide sex-specific therapeutic interventions in obesity.

**Supplementary Information:**

The online version contains supplementary material available at 10.1186/s13293-026-00859-z.

## Abstract (plain language summary)

Estrogen is a sex hormone that plays critical roles in both women and men, regulating not only reproduction but also how the body stores and uses fat. However, how individual types of cells respond to estrogen in females versus males remains unknown. Here, we analyzed how estrogen treatment affects individual cells and genes in visceral fat, the adipose depot surrounding internal organs and is tightly linked to metabolic diseases, in obese female and male mice. Our findings revealed that among the adipose cell types, stem-like cells that can form new fat cells and a specific type of immune cells called macrophages were particularly sensitive to estrogen in both sexes. Estrogen also caused more pronounced changes in females than in males, affecting both the number and the gene expression patterns of these cells. In female fat tissue, estrogen appeared to maintain more stem cells and reduce genes involved in fat cell differentiation and extracellular matrix remodeling. In macrophages of both sexes, estrogen lowered inflammation, though female mice showed additional fine-tuned control of immune and energy-related pathways and enhanced communication between adipose stem cells and macrophages. Lastly, estrogen-responsive genes were also linked to human metabolic disease/traits such as cholesterol levels, diabetes, and heart disease risks. Collectively, our findings support that estrogen shapes visceral fat tissue in a sex- and cell-type specific manner and point to key cell types and biological processes underlying differences in metabolic health and risk between men and women.

**Supplementary Information:**

The online version contains supplementary material available at 10.1186/s13293-026-00859-z.

## Highlights

- Estradiol reduced fat accumulation in both sexes, with a stronger effect in female mice.
- Single-cell analyses identified adipose stem and progenitor cells (ASPCs) and macrophages in gonadal adipose tissue as the most estrogen-responsive cell types, with prominent changes in cell composition and gene expression.
- Estrogen altered key biological pathways in a sex- and cell-type dependent manner by enhancing stemness, energy metabolism, and protein synthesis in female ASPCs and modulating immune and metabolic pathways in female macrophages, while broadly reducing inflammation in both sexes.
- Estrogen enhanced macrophage to ASPC communication in females and estrogen-responsive genes showed sex-specific association with metabolic diseases, providing insight into sex differences in obesity and metabolic disease risk and treatment.

**Supplementary Information:**

The online version contains supplementary material available at 10.1186/s13293-026-00859-z.

## Background

Obesity is a highly prevalent and complex metabolic disorder characterized by the excessive buildup of body fat, which markedly increases the risk of Type 2 diabetes (T2D), metabolic dysfunction-associated steatotic liver disease (MASLD), hypertension, coronary artery disease, stroke, and certain cancers, creating a substantial economic and health burden [1, 2]. A central feature of obesity is pathological expansion of white adipose tissue (WAT), particularly the visceral fat, which is strongly associated with heightened cardiometabolic risk [3] due to its higher reliance on hypertrophy during expansion, its proximity to vital organs, and its capacity to release pro-inflammatory adipokines and free fatty acids directed toward other organs [4, 5]. Consequently, reducing visceral adipose tissue represents a promising therapeutic strategy to improve cardiometabolic health.

Sex differences in white adipose tissue are evident in both humans and mouse models. Females generally have smaller visceral adipocytes than males, and males preferentially accumulate excess fat in visceral depots [6, 7]. These sex-biased traits contribute to distinct metabolic outcomes, as males typically exhibit greater adipose inflammation and immune cell infiltration, leading to higher cardiometabolic risk compared with females [8–10]. Among factors driving such sex differences, sex steroids play a significant biological role. Healthy females in reproductive years have higher levels of estradiol (E2), the primary estrogenic hormone that is generally considered protective, compared to males and post-menopausal females. Protective effects of exogenous E2 treatment on fat distribution, glucose metabolism, and insulin sensitivity have also been reported in menopausal women and men receiving E2-related hormone therapy as well as in mouse and other animal models [11–13]. While E2-estrogen receptors (ERs) signaling has been shown to regulate adipogenesis partially through autophagy in visceral adipocytes in a female-biased manner [14], how E2 influences broader aspects of adipose remodeling, including transcriptional regulation in diverse cell types and cell–cell interactions, particularly in a sex-specific manner, remains to be explored [15].

Using single-cell transcriptomics, here we examine how exogenous E2 treatment shapes gene expression and regulatory networks across adipose progenitor cells, macrophages, and other stromal cell types in gonadal white adipose tissue (gWAT), a key visceral adipose depot, from gonadectomized XX and XY mice with diet-induced obesity. By delineating sex-specific cell types, pathways, and molecular and cellular interactions sensitive to E2 treatment, our findings provide insights into the cell type-independent *vs.* cell type-specific regulatory role of estrogen in visceral adipose tissue to better understand sex differences in obesity risk and treatment to facilitate personalized therapeutic strategies for both sexes.

## Methods

### Animal and dietary treatment

C57BL/6 J mice from the Jackson Laboratory were delivered at 8 weeks of age. Mice (n = 6–8/sex/treatment) were housed at 22 ± 1 °C in a 12:12-h light–dark cycle and fed chow diet ad-libitum. At 9 weeks of age, all mice received gonadectomy. Animals were anesthetized under inhaled isoflurane (1–5% in 100% oxygen, 1–2 L/min). 2% chlorhexidine solution was used as antiseptic to clean the surgical site prior to incision. In biological males, midline scrotal excision was used to externalize tunica. An additional incision was made to the tunica to expose the testes and vas deferens. The vas deferens was cauterized, and testes were removed. In biological females, a midline dorsal skin incision beginning at the midthoracic curvature was followed by lateral incisions to the dorsal peritoneal wall to visualize each ovary. The oviduct was exteriorized and cauterized, and the ovary was removed. Prophylactic antibiotic was given once postoperatively (ceftriaxone, 15–25 mg/kg) and analgesic once preoperatively and every 24 h postoperatively for 3 days (ketoprofen, 5–10 mg/kg). Following a three-week surgical recovery, mice were transferred to a 60% kcal High Fat Diet (HFD, Research Diets, D12492) ad-libitum to induce obesity prior to estradiol treatment. Treatment groups were as follows: XY + E2 (XYE), XY + Placebo (XYV), XX + E2 (XXE), and XX + Placebo (XXV). Starting at week 33, intraperitoneal injection of 2 μg of 17β-estradiol-3-benzoate (E2) (Sigma Aldrich #E8515) in sesame oil, or sesame oil alone as vehicle treatment, was given every four days to mice of both sexes through Week 47, based on prior studies which demonstrated physiological effects [16–18]. Mice were sacrificed and tissues were collected at the end of Week 47. Due to limited access to laboratory resources during the COVID pandemic, body weight was measured at Week 31 prior to E2 treatment. Weekly body weight measurements started from Week 33 (beginning of E2 treatment) to Week 47. Similarly, NMR measurements were first taken at Week 30, then at Week 40 as post-treatment measurement. Estradiol was assayed using 10% EDTA anticoagulated serum at Week 47, with analysis performed via ELISA (Abcam #108667) using the protocol described in Iijima et al. [19]. Circulating coagulation factors were assessed at Week 47 using 3% sodium citrate anticoagulated serum via the following ELISA kits: coagulation factor 7 (NOVUS Biologicals #NBP2-67963), coagulation factor 8 (NOVUS Biologicals #NBP2-76578), and coagulation factor 10 (NOVUS Biologicals #NBP2-82404).

### Tissue dissociation and single cell preparation

Gonadal white adipose tissue was dissociated and single cells from stromal vascular fraction (SVF) were isolated using previously optimized protocol [20, 21]. Briefly, gWAT was minced and transferred to 2 mL of digestion buffer (PBS with Collagenase II at 3 mg/ml; Worthington Biochemical, Lakewood, NJ, USA), then incubated at 37 °C for 40 min. In the end of incubation, 8 mL of medium (DMEM with glutamax supplemented with 10%FBS and 1% pen/strep; Thermo Scientific, CA) was added to stop enzyme activity. The digestion mixture was passed through a 100 μm cell strainer and centrifuged. The top layer containing mature adipocytes was removed and the remaining SVF cell suspension was centrifuged at 150 × g for 8 min. The resulting pellet was resuspended in RBC lysis buffer (Thermo Scientific, CA) to lyse the red blood cells, centrifuged, resuspended in DMEM medium, and spun down again. Finally, the cell pellet was resuspended in freezing medium (FBS containing 10% DMSO) and cryopreserved until use.

For the single cell assay, the cryopreserved cell suspension was thawed using dropwise thawing method from 10 × Genomics. Following thawing and washing, the resulting cell pellet was resuspended in 1 mL of 0.04% BSA/PBS solution, and filtered through a 40 µm Flowmi Cell Strainer (Fisher Scientific, Hampton, NH, USA) to remove cell aggregates and debris. Cells were centrifuged and the final cell pellet was resuspended in 0.04% BSA/PBS, yielding final concentration of 700–1200 cells/μL. Cell viability was assessed by trypan blue staining, with all samples showing over 85% viability.

### scRNAseq data generation and preprocessing

scRNAseq was conducted following the 10 × 3′ single-cell RNA-seq V3.1 protocol (10 × Genomics, Pleasanton, California, USA). For each group, n = 3 independent biological replicates were processed, and a total of 12 independent libraries were constructed (3 replicates × 2 treatment × 2 sexes = 12). The concentrations of cDNA and library were measured using the Qubit Fluorometric Quantitation method (Thermofisher Scientific, Waltham, MA, USA), and their qualities were evaluated using the Agilent 2200 TapeStation system (Agilent, Santa Clara, CA, USA). The 12 libraries were sequenced on the Novaseq S4 platform with 2 × 100 bp paired-end reads (Illumina, San Diego, CA, USA) in the UCLA Broad Stem Cell Research Center, achieving ~ 25 k reads/cell. Fastq files were generated using the mkfastq function on the Cell Ranger software version 5.0.1 (10 × Genomics, Pleasanton, California, USA)**.** The 10X Genomics CellRanger (v7.1.0) [22] was further used for individual sample demultiplexing, sequencing alignment to *Mus musculus* genome assembly (mm10), barcode and unique molecular identifier (UMI) counting, and feature quantification to generate gene count matrices. Output filtered feature-barcode matrix files from CellRanger were analyzed using the Seurat R package (v4.4.0) [23]. Single cells were selected based on the number of detected genes (200 to 7,000), number of UMIs (< 20,000), and the proportion of mitochondrial reads (< 10%).

### Cell clustering and cell type annotation

Feature counts for each cell were divided by the total counts for that cell, multiplied by the scale factor of 10,000, and log transformed. Standard processing was performed using Seurat functions *FindVariableFeatures*, *ScaleData*, and *RunPCA* with default parameters. The first 30 principal components (PCs) from Principal Component Analysis were used for KNN graph construction with *FindNeighbors*, followed by *FindClusters* to cluster cells with Leiden clustering. Uniform Manifold Approximation and Projection (UMAP) was used to visualize single-cell data at two dimensions. Marker genes of each cluster were identified using the Wilcoxon rank sum test through *FindAllMarkers* function. Cell type identities were determined based on the comparison of cluster markers with canonical cell-type-specific marker genes and previous scRNAseq studies on mouse visceral adipose SVF [24–27]. Doublet detection was performed using the DoubletFinder R package (v2.0.4) [28]. After the removal of predicted doublets, cell fraction analysis was conducted using two-way analysis of variance (ANOVA) followed by post hoc pairwise comparisons with TukeyHSD.

### Euclidean distance for cell type transcriptional sensitivity

To quantify and compare the magnitude of transcriptional responses to E2 treatment across cell types and sex chromosome backgrounds, we performed a Euclidean distance analysis [29]. We first identified the top 2,000 highly variable genes (HVGs) across the full dataset using Seurat’s *FindVariableFeatures* function with the variance-stabilizing transformation (VST) method. For each cell type with ≥ 10 cells per condition, we computed Euclidean distances between average expression profiles of treatment groups (XXE vs. XXV; XYE vs. XYV) using the HVGs after z-score normalization. To assess significance, we generated null distributions from 5,000 permutations in which cell labels were randomly reassigned between groups while preserving original group sizes. Empirical *p*-values were defined as the proportion of permuted distances greater than or equal to the observed distance, and adjusted for multiple testing using the Benjamini–Hochberg false discovery rate (FDR) method. Effect sizes were quantified as log fold changes (logFC), defined as log10(observed distance) – log10(median permuted distance), with positive values indicating stronger-than-expected transcriptional shifts. Significance was defined at FDR < 0.05.

### Differential gene expression analysis

To determine genes affected by E2 treatment, we compared the transcriptome of each cell population between vehicle treatment group and estradiol treatment group for XX and XY mice, respectively, using a Wilcoxon rank sum test through *FindMarkers* function in Seurat. Genes expressed in at least 10% of the cells in each cell type in either of the comparison groups (XXE vs. XXV or XYE vs. XYV) were considered and genes passing Bonferroni-adjusted *p*-value < 0.05 were considered significant differentially expressed genes (DEGs). *Gm42418* and *AY036118* were excluded from the DEG results since they were found to be driven by technical biases instead of true biology [30–32]. DEGs were ranked by a priority score combining log fold change and statistical significance, calculated as |avg.log₂FC|× (-log₁₀(*p.adj* + 1 × 10⁻^3^⁰⁰)), with the small constant (1 × 10⁻^3^⁰⁰) added to prevent computational errors when dealing with extremely significant *p*-values approaching zero. UpSet plots were generated to visualize overlapping and unique DEGs between E2-responsive DEGs in XX and those in XY within each cell type using the UpSetR R package (v 1.4.0) [33]. Genes were selected from the top 10 genes with the highest priority scores as representative genes for that intersection.

### Stratified rank–rank hypergeometric overlap (RRHO) analysis

To assess the concordance of estrogen responses between sexes across adipose cell types, we performed stratified RRHO analysis with RRHO2 R package (v 1.0) [34]. For each cell type, unfiltered differential expression results from the two comparison groups (XXE vs. XXV and XYE vs. XYV) were used to construct ranked gene lists. Each gene was assigned a signed significance score based on -log10(pvalue) * sign(effectSize), where upregulated genes received positive scores and downregulated genes received negative scores. The two lists were restricted to the set of shared genes, which were compared for concordance or discordance in their ranks between sexes and visualized using *RRHO2_heatmap*.

### Pathway enrichment analysis and visualization

To identify enriched pathways, we performed hypergeometric tests on our cell type, sex, and direction-specific DEGs against the mouse-specific Gene Ontology (GO) Biological Process database [35] with multiple testing corrected with the Bonferroni method. The enrichment score was calculated as Gene Ratio (i.e., overlapped gene count divided by DEG gene count) divided by Background Ratio (i.e., specific pathway gene count divided by the total number of genes detected in the study). Significant pathways were selected based on p.adj < 0.01 and the number of overlapped genes ≥ 20, and visualized using the ggplot2 R package (v 3.4.4) [36].

### Transcription factor (TF) enrichment analysis

To identify potential transcription factors mediating the effects of E2 treatment on DEGs, we performed TF Enrichment Analysis on cell-type- and sex-specific DEGs with Enrichr [37]. TF-target relations were derived from ChEA2022 [38], which includes curated ChIP-seq, ChIP-chip, and other transcription factor binding site profiling studies. Significantly enriched transcription factors (*p.adj* < 0.05) were further checked for overlap with ERɑ and ERβ mouse ChIP targets from ChEA2022 to sort out direct versus indirect regulation of DEGs by E2.

### Single-cell gene regulatory network (GRN) construction and weighted key driver analysis

To identify additional cell-type-specific regulators mediating the E2-associated DEG alterations, we applied SCING [39], a gradient boosting machine learning approach, to our scRNAseq raw count matrix data to construct cell-type-specific intracellular GRNs. Key Driver Analysis (KDA) was then performed with KDA functions from the Mergeomics pipeline [40]. In brief, KDA maps sex-, cell-type-, and direction-specific DEGs onto the corresponding cell-type-specific GRNs constructed by SCING and uses a χ2-like statistic to identify key drivers (KDs) that are connected to a significantly larger number of DEGs than what would be expected by random chance. Significant KDs were selected at FDR < 0.05, and subnetworks associated with the top significant KDs were visualized using Cytoscape [41].

### Cell–cell communication analysis

CellChat (v 2.1.2) [42] was used to evaluate the effects of E2 treatment on cell–cell communications in XX and XY groups. Epithelial cells and LECs were excluded from the analysis due to low cell counts. Intercellular networks were constructed for each genotype (XXV, XXE, XYV, XYV) and network changes in response to E2 were derived based on differences in the number and strength of interactions among cell populations between vehicle and E2 groups for each sex. Upregulated and downregulated ligands and receptors were determined in each cell group through differential expression analysis with the *identifyOverExpressedGenes* function. Finally, altered Ligand-Receptor pairs across cell populations were visualized using the *netVisual_chord_gene* function.

### Connecting sex- and cell-type-specific DEGs with human metabolic traits and diseases

To evaluate the relevance of E2-induced DEGs to human health, we obtained summary statistics for 85 publicly available GWAS traits and diseases from the GWAS catalog [43]. For each trait/disease, SNP redundancy was reduced using Marker Dependency Filtering (MDF) from the Mergeomics pipeline [40], where SNPs were pruned at 70% linkage disequilibrium. SNP-to-gene mapping was performed using adipose visceral omentum eQTL data from human GTEx (V8) [44]. These human genes were then converted to mouse orthologs for comparison with our E2-induced cell-type-specific DEGs. Marker Set Enrichment Analysis (MSEA) from Mergeomics was then used to test whether SNPs associated with our sex-, cell type-, and direction-specific DEGs showed stronger trait/disease association compared to SNPs linked to random gene sets, using a χ2-like statistic. Significant DEG-trait enrichments were determined by FDR < 0.05.

### Statistical analysis

All statistical analyses were conducted in R (v4.3.2). Prism (v10.5.0) was used to visualize phenotypic measurements and cell fraction analysis results. For comparisons involving sex (XX, XY) and treatment (vehicle, E2), two-way analysis of variance (ANOVA) model was used to test main and interaction effects. Post hoc pairwise comparisons among combined sex × treatment groups were performed using Tukey’s multiple comparison test. For each ANOVA term, we report the *F* statistic, degrees of freedom, *p*-value, and partial eta squared (η2ₚ) as the measure of effect size. Multiple testing correction across cell types was performed using the Benjamini–Hochberg FDR method. Statistical significance was defined as FDR or *p.adj* < 0.05 unless otherwise stated. All values are presented as mean ± SEM.

## Results

### E2 treatment lowered body weight and fat mass in both XX and XY obese mice

**Fig. 1:**
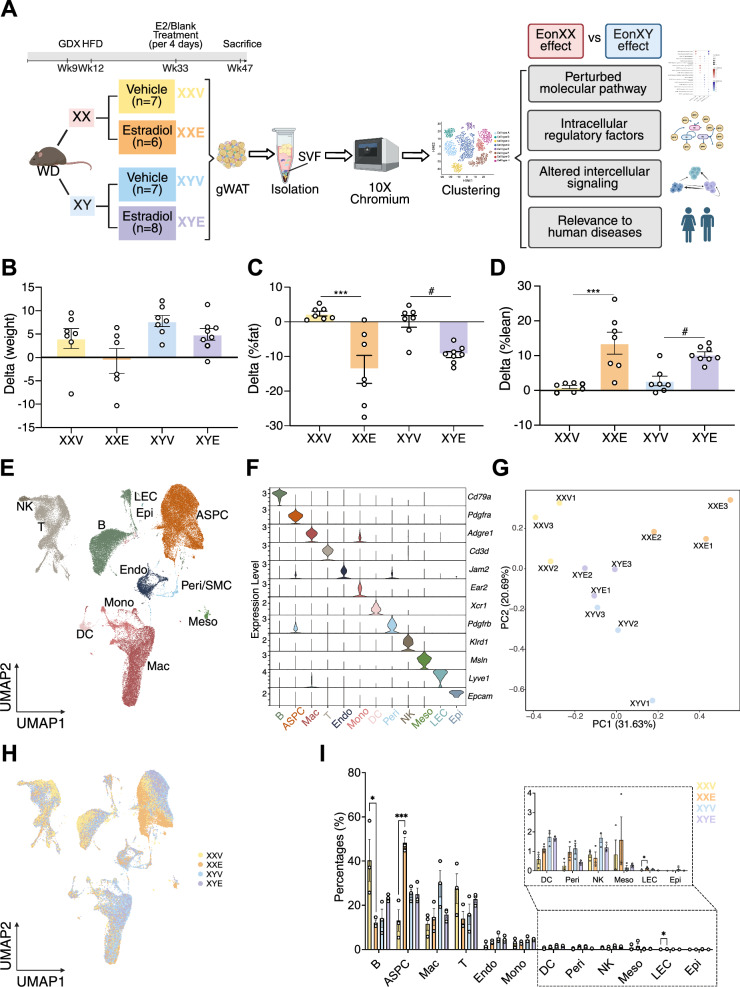
Phenotypic measurements and single-cell atlas of gonadal adipose SVF in diet-induced obese, gonadectomized mice. (**A**) Experimental workflow and single-cell RNA-seq of mouse gWAT. (**B**) Body weight change across four groups of mice at Week 47, with baseline measurements at Week 31 (n = 6–8/sex/treatment). (**C**) Changes in fat mass percentage calculated via NMR across groups of mice post-E2 at Week 40 relative to week 30 baseline measurements (n = 6–8/sex/treatment). (**D**) Changes in lean mass percentage across groups of mice at Week 40 vs baseline (n = 6–8/sex/treatment). (**E**) Uniform manifold approximation and projection (UMAP) of clusters comprising 65,843 mouse SVF cells in gWAT (n = 3/sex/treatment). (**F**) Violin plots showing the expression of canonical marker genes across annotated cell types. (**G**) Principal component analysis (PCA) on the top 2,000 highly variable genes across all mice. (**H**) UMAP projection of clusters colored by experimental groups. (**I**) Analysis of cell fraction using Two-way ANOVA followed by post hoc tests with TukeyHSD. Statistical significance was shown only for XXE.v.XXV and XYE.v.XYV comparisons. * *p* < 0.05, ** *p* < 0.01, *** *p* < 0.001 in XX mice (XXE vs XXV); ^#^ *p* < 0.05 in XY mice (XYE vs XYV)

To examine the effects of estradiol on the metabolic health of XX and XY mice, gonadectomized C57BL/6 XX and XY mice (Fig. 1A) were fed with a HFD to induce obesity and then subject to subcutaneous injection of 2 μg of 17β-estradiol (E2) (indicated XXE or XYE), or vehicle treatment (indicated XXV or XYV), once every four days for 3 months while on HFD (n = 6–8/sex/treatment). We confirmed that E2 treatment led to elevated levels of circulating E2 (Fig. S1A), lower weight gain in response to HFD, and reductions in body fat percentages and increases in lean mass percentage in both sexes, with XX mice on E2 treatment showing stronger phenotypic changes compared to XY mice (Fig. 1B-D, Fig. S1B-C). Since female hormone replacement therapy has been reported to be associated with an elevated risk of thromboembolism [45, 46], circulating levels of coagulation factors 7, 8, and 10 were also measured. However, no significant changes were observed in either sex group (Fig. S1D-F).

### E2 induced stronger gWAT transcriptomic and cell proportion shifts in XX than in XY mice

To elucidate the molecular programs altered by E2 treatment in adipose tissue at single-cell resolution, we carried out scRNAseq on the SVF from gWAT of mice from each group (n = 3 independent tissues/group/sex for a total of 12 samples) (Fig. 1A). After quality control (Fig. S2A), we retained 65,843 high quality cells and identified 12 cell types based on both canonical markers and reference markers from previous studies [24–27]: adipose stem and progenitor cells (ASPC), B cells (B), T cells (T), natural killer cells (NK), macrophages (Mac), dendritic cells (DC), monocytes (Mono), endothelial cells (Endo), mesothelial cells (Meso), pericytes (Peri), lymphatic endothelial cells (LEC), and epithelial cells (Epi) (Fig. 1E-F, Fig. S2B).

Principal component analysis (PCA; Fig. 1G) across samples showed that 31.63% of the transcriptomic variation was explained by E2 treatment, especially in the XX groups (XXE vs. XXV in PC1), suggesting that the major variation came from the E2 effect on XX mice. Around 20% of the transcriptomic variation was explained by sex chromosome complements (XX vs. XY in PC2). XY mice with E2 treatment also had a transcriptomic shift towards XX mice, indicating that E2 altered the XY transcriptome to resemble the profile of XX mice without E2 treatment.

UMAP visualization of individual cell types showed that cells from the majority of cell types aligned well across the four groups of mice, but an evident shift was observed in ASPCs in the XX mice following E2 treatment (Fig. 1H, Fig. S2C). To quantify transcriptomic shift in individual cell types, we performed a Euclidean distance-based analysis on the top 2,000 highly variable genes (HVGs, details in Methods). This quantitative method confirmed that E2 induced significant transcriptomic changes (in the form of log2 fold change) across most cell types (except epithelial cells and LECs due to low cell count) in each sex, although the magnitude of responses (i.e., log2 fold changes) was stronger in XX than in XY mice (Fig. S2D). In both sexes, significant transcriptomic shifts were detected in ASPCs, B cells, macrophages, endothelial cells, dendritic cells, mesothelial cells, and T cells.

In addition to the sex-biased global and cell-type-specific transcriptomic shifts, E2 treatment also altered the proportions of several adipose cell types. Two-way ANOVA revealed a significant main effect of E2 treatment (*F*(1,8) = 29.04, *p* = 0.0007, FDR = 0.0118, η2ₚ = 0.78) and a significant sex × treatment interaction (*F*(1,8) = 30.08, *p* = 0.0006, FDR = ^0.0118, η2^ₚ = 0.79) for ASPCs, indicating sex-dependent responsiveness to E2 (Fig. 1I**; **Fig. S2E). Post hoc Tukey comparisons confirmed that E2 significantly increased ASPC fractions in XX mice (*p.adj* = 0.019), but not in XY mice. Significant sex × treatment interactions were also observed in LECs (*F*(1,8) = 15.83, *p* = 0.004, FDR = 0.0366, η2ₚ = 0.66). Collectively, these results indicate that E2 treatment remodeled adipose cellular composition, particularly in females.

### E2 treatment shifted ASPC and macrophage subtypes

We further identified ASPC and macrophage subtypes to investigate whether E2 alters cellular subpopulations important for obesity [47, 48]. Three ASPC subtypes were identified based on the reference marker genes [26, 49]: adipose-derived stem cell (ASC), preadipocyte (preA), and adipogenesis regulators (Areg) (Fig. S3A-B). E2 induced shifts in the transcriptional patterns in the XX group, especially in ASC and preA populations (Fig. S3C-D). Cell proportion analysis showed non-significant trends for increased ASC and decreased preA, with the effect more apparent in XX than in XY mice (Fig. S3E). The decrease in preadipocytes may lead to fewer adipocytes, which may explain the greater fat mass percentage reduction in XX mice by E2 treatment (Fig. 1C).

For macrophages, five subtypes were identified based on reference marker genes [25, 50, 51], including perivascular-like macrophage (PVM), non-perivascular-like macrophage (NPVM), lipid-associated macrophage (LAM), proliferating LAM (P-LAM), and collagen-expressing macrophage (CEM) (Fig. S4A-B). We observed a transcriptional pattern shift in the PVM population of XX mice (Fig. S4C-D) as well as an increasing trend in PVM proportion and a decreasing trend in LAM proportion in both sexes (Fig. S4E) after E2 treatment. These trends in macrophage subpopulations support a beneficial profile as increasing LAM and decreasing PVM are associated with obesity and metabolic disorders [52, 53].

### Concordance and discordance of E2-induced gene expression changes between sexes

**Fig. 2:**
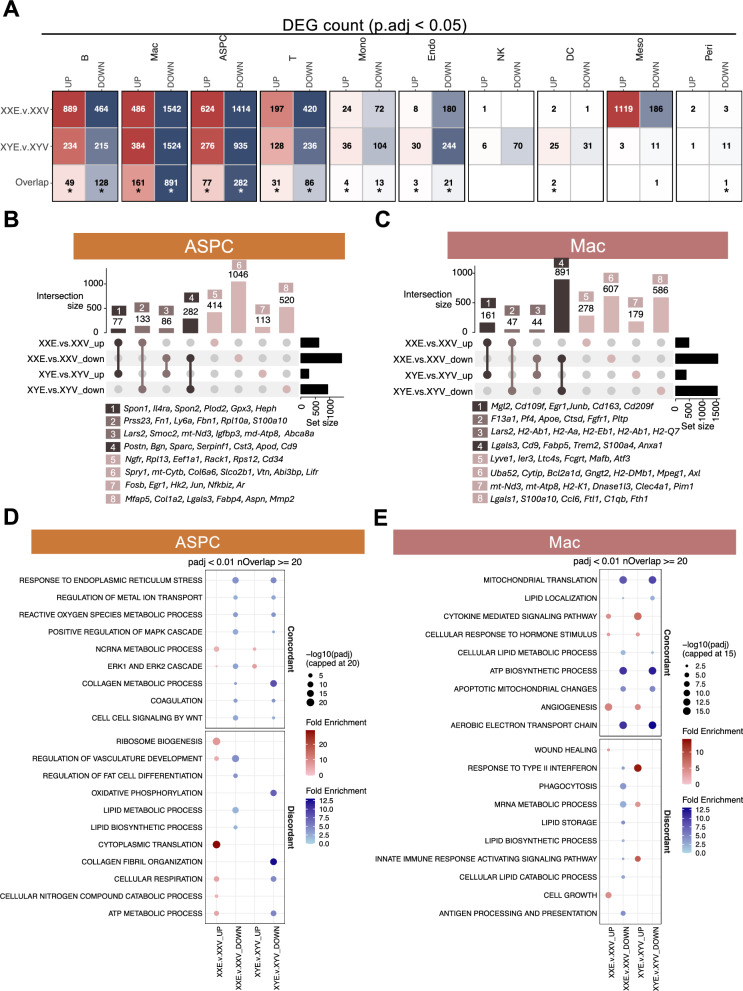
Sex-Specific concordance and discordance in transcriptomic responses to E2 treatment in mice. (**A**) Number of sex-specific and shared differentially expressed genes (DEGs) at FDR < 5% across adipose cell types. (**B-C**) UpSet plots showing E2 effects on ASPCs (**B**) and macrophages (**C**) by regulation direction in XX and XY mice. (**D-E**) Pathway analysis highlighting sex-concordant and discordant pathways enriched in ASPCs (**D**) and macrophages (**E**) in response to E2 treatment at FDR < 1% and number of enriched DEGs ≥ 20. Red dots represent pathways enriched with upregulated DEGs in the respective comparison, whereas blue dots represent pathways enriched with downregulated DEGs. For visualization purposes, dot sizes, which represent -log_10_(*p.adj*) value of the pathway, were capped at 20 for ASPCs and at 15 for macrophages

We identified DEGs induced by E2 in individual gWAT cell types at FDR < 5% (Table S1). As shown in Fig. 2A, the largest numbers of DEGs were found in ASPC and macrophage populations in both XX and XY mice, followed by other immune cell types such as B cell and T cell, where most of the DEGs were downregulated by E2 treatment and more DEGs were found in XX than in XY mice. Significant overlaps were found between the two sexes for most cell types, indicating the conserved effects of E2 on gene regulation regardless of sex. However, E2 also induced a unique, majorly up-regulation of genes in mesothelial cells in XX mice (1119 upregulated among1305 total DEGs) but not in XY mice (3 upregulated among 14 total DEGs).

To further assess concordance and discordance of E2-induced transcriptomic changes between sexes on a global level accounting for directional effects, we applied RRHO to the fold changes of all genes, regardless of statistical significance (details in Methods). Strong global concordance in gene regulation between sexes was observed in most cell populations (Fig. S5A-J, Fig. S5K), including ASPC (Fig. S5A) and macrophages (Fig. S5B), particularly among genes downregulated by E2. In contrast, strong discordance between sexes was detected in mesothelial cells (Fig. S5C) and dendritic cells (Fig. S5J). These results indicate that the degree of concordance in E2 regulation varies by cell type.

### Concordant and discordant DEGs and pathways regulated by E2 in ASPCs and macrophages

As ASPCs and macrophages had the largest numbers of DEGs, we focused on these two cell types to further examine the consistency *vs.* differences in E2-induced DEGs between sexes, where DEGs at FDR < 5% were categorized as either concordant (regulated in the same direction in both sexes) or discordant (regulated in opposite directions or in one sex only).

In ASPCs, the majority of E2-induced DEGs were up- and downregulated exclusively in one sex (Fig. 2B). Among genes uniquely downregulated in XX mice (bar 6), adipogenesis and progenitor cell stemness-related genes *Spry1* (Sprouty1) and *Lifr* (Leukemia inhibitory factor receptor) ranked at the top. Genes uniquely upregulated in XX mice (bar 5) included stemness-related genes *Ngfr*, *Cd34*, and ribosomal/translational regulators *Rpl13*, *Eef1a1*, *Rack1*, and *Rps12*. Genes consistently downregulated in both sexes (bar 4) included ECM-related genes *Postn*, *Bgn*, and *Sparc*. XY-specific downregulated genes included ECM (*Col1a2*, *Aspn*, *Mmp2*) and fibrosis-related genes (*Lgals3*, *Fabp4*, *Mfap5*).

Since obesity is often associated with maladaptive ECM remodeling in adipose tissue, characterized by excessive and pathological deposition of matrix components [54–56], we further examined the DEGs related to ECM components as well as enzymes involved in ECM synthesis and degradation in both sexes across ASPC subtypes (Fig. S6). E2 downregulated these genes predominantly in the preadipocyte population, where genes encoding collagens, glycoproteins, matrix metalloproteinases (MMPs), and tissue inhibitors of metalloproteinases (TIMPs) were mainly downregulated in XY mice, with fewer genes affected in XX mice. In contrast, more genes related to proteoglycans, whose functions are associated with inflammation modulation under obesity [57–59], were downregulated in XX mice compared to XY mice, suggesting sex-biased targets of E2 treatment at ECM components and related enzymes.

At pathway level (Fig. 2D**; **Table S2), non-coding RNA metabolic process was concordantly upregulated, with a higher significance level in XX mice. Response to endoplasmic reticulum stress and collagen metabolic process were concordantly downregulated. Discordant pathways included XX-specific upregulation of ribosome biogenesis and cytoplasmic translation, XX-specific downregulation of fat cell differentiation, lipid metabolic process, and lipid biosynthetic process, XY-specific downregulation of collagen fibril organization, and XX-specific upregulation and XY-specific downregulation of cellular respiration and ATP metabolic process.

In macrophages, the largest subset of DEGs comprised 891 genes concordantly downregulated in both sexes, followed by discordant downregulation of 607 genes in XX mice and 586 genes in XY mice (Fig. 2C). Concordantly downregulated genes (bar 4) included LAM marker genes, *Trem2* and *Cd9*, as well as pro-inflammatory macrophage activity genes, such as *Lgals3*, *Fabp5*, and *S100a4*. XY-specific downregulated genes (bar 8) include ones central to macrophage inflammatory and metabolic functions (*Lgals1*, *Ccl6*, *Ftl1*, and *Fth1*). Genes downregulated in XX but upregulated in XY included *Lars2*, *H2-Ab1*, *H2-Aa*, *H2-Eb1*, and *H2-Q7*, which are associated with functions such as energy production support, antigen presentation, and immune modulation.

Pathway analysis of sex- and direction-stratified DEG sets revealed further contrasts (Fig. 2E**;** Table S2). Concordantly downregulated pathways in both sexes included mitochondrial translation, ATP biosynthetic process, apoptotic mitochondria changes, aerobic electron transport chain, and lipid metabolism-related processes. In contrast, cytokine-mediated signaling pathway, cellular response to hormone stimulus, and angiogenesis were concordantly upregulated. Pathways showing opposite regulation between sexes included response to type II interferon, mRNA metabolic process, and innate immune response-activating signaling pathway, all of which were downregulated in XX but upregulated in XY. In XX mice only, phagocytosis, lipid storage and biosynthetic process, and antigen processing and presentation were downregulated, while wound healing and cell growth pathways were uniquely upregulated.

### E2 induces conserved transcription factor (TF) networks in ASPCs and macrophages relevant to estrogen signaling and additional sex-specific TF networks

**Fig. 3:**
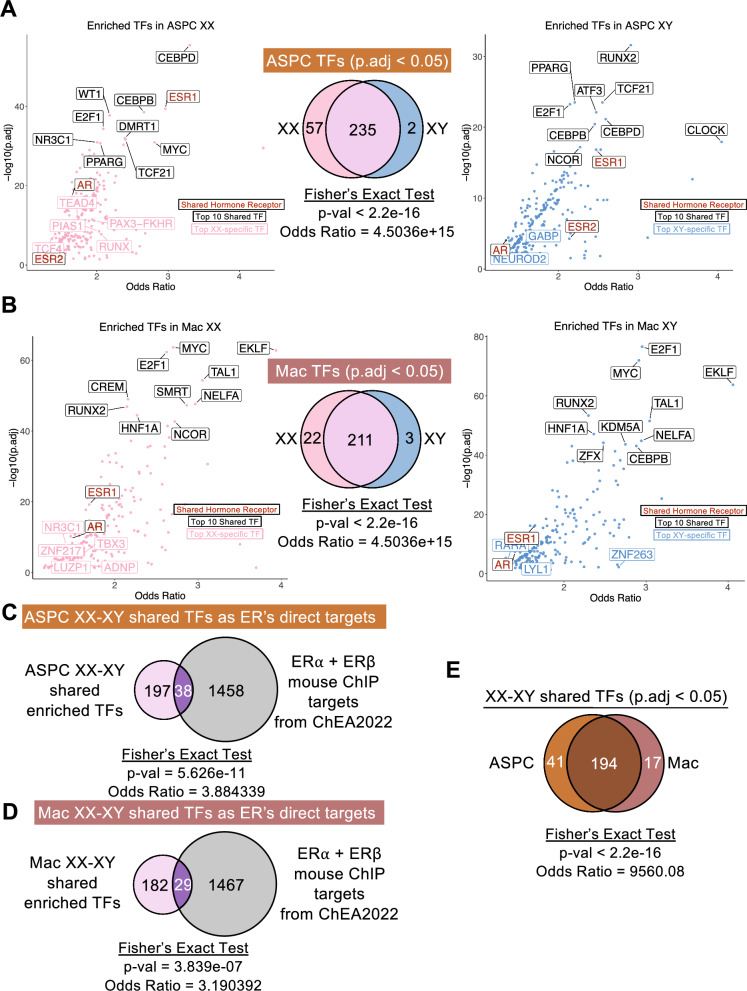
Transcription factor (TF) enrichment analysis on E2-associated DEGs in ASPCs and macrophages of XX and XY mice. (**A-B**) Enriched TFs in ASPCs (**A**) and macrophages (**B**) for XX (left) and XY (right) mice, highlighting selected shared hormone receptors, the top 10 shared TFs, and the top five (if any) sex-specific TFs. (**C-D**) Overlap of XX-XY shared enriched TFs in ASPC (**C**) and macrophage (**D**) with known direct ERα and ERβ targets in mice. (**E**) Overlap of XX-XY shared enriched TFs between ASPC and macrophage

To dive into the transcriptional regulatory cascade behind hundreds to thousands of DEGs in response to E2 treatment, especially in ASPCs and macrophages, we performed TF enrichment analysis using cell-type-specific DEGs for the two sex groups. In ASPCs, the majority (235) of the enriched TFs were shared between XX and XY, whereas 57 were unique to XX mice and three were specifically enriched for DEGs in XY mice (Fig. 3A). These results suggest that TF-mediated E2 signaling is largely conserved between sexes in ASPCs. Among the consistent TFs, hormone receptors such as ESR1, ESR2, and AR were enriched, supporting the importance of ER signaling in E2 actions. Additionally, among the 235 shared TFs between sexes, 38 TFs were known downstream targets of ER signaling in mouse tissues based on ChEA2022 [38], further supporting the essential role of ER signaling. Additional top TFs in ASPC shared between sexes include CEBPD, CEBPB, E2F1, TCF21, and PPARG, which are regulators of adipogenic and metabolic pathways, adipose progenitor cell differentiation, cell cycle, and mesenchymal identity, reinforcing the role of E2 in influencing ASPC proliferation and lineage commitment.

Among the sex-biased TFs in ASPCs, XX-specific TFs include TEAD4, TCF4, and RUNX, which are regulators of stem cell maintenance, mesenchymal lineage determination, and adipogenesis regulation. On the other hand, XY-specific TFs GABP and NEUROD2 suggest that XY ASPCs engage mitochondrial and metabolic regulation in response to E2.

In macrophages, we observed a similar strong concordance in TF enrichment pattern between the two sexes, with 211 shared TFs, 22 XX-specific and three XY-specific TFs (Fig. 3B). ESR1 and AR, but not ESR2, were among the shared TFs between sexes. Other top concordant TFs between sexes include MYC, EKLF, E2F1, RUNX2, NELFA, HNF1A, and TAL1, which are critical for macrophage activation, differentiation, and metabolism.

Among the top XX-specific TFs in macrophages are corepressors SMRT (Silencing Mediator for Retinoid and Thyroid Hormone Receptors) and NCOR (Nuclear Receptor Co-repressor 1), which are known to repress inflammatory pathways such as NF-κB (Fig. 3B). Other top XX-specific TFs include NR3C1 (Glucocorticoid Receptor, GR), ZNF217 (chromatin remodeling and metabolic control), TBX3, LUZP1, and ADNP, which are related to inflammation and macrophage differentiation and remodeling. In contrast, only three TFs, ANF263, RARA (cholesterol efflux and inflammation), and LYL1 (a hematopoietic TF), were uniquely enriched in XY macrophages.

Notably, approximately 17% (38/235) of sex-shared TFs in ASPCs and 14% (29/211) in macrophages were known direct ER targets (Fig. 3C-D), suggesting direct effect of E2 on these cell types via ER signaling. Most sex-shared TFs were also consistent between ASPCs and macrophages, supporting the presence of core ER-dependent and independent transcriptional regulators that operate across cell types in response to E2 (Fig. 3E).

### E2 induces sex-specific and conserved gene regulatory networks (GRNs) in ASPCs and macrophages

In addition to the TF-focused network analysis above, we also identified cell type-specific regulators by constructing data-driven GRNs to identify additional network key driver (KD) genes whose network neighborhoods were significantly enriched with E2-responsive genes.

**Fig. 4:**
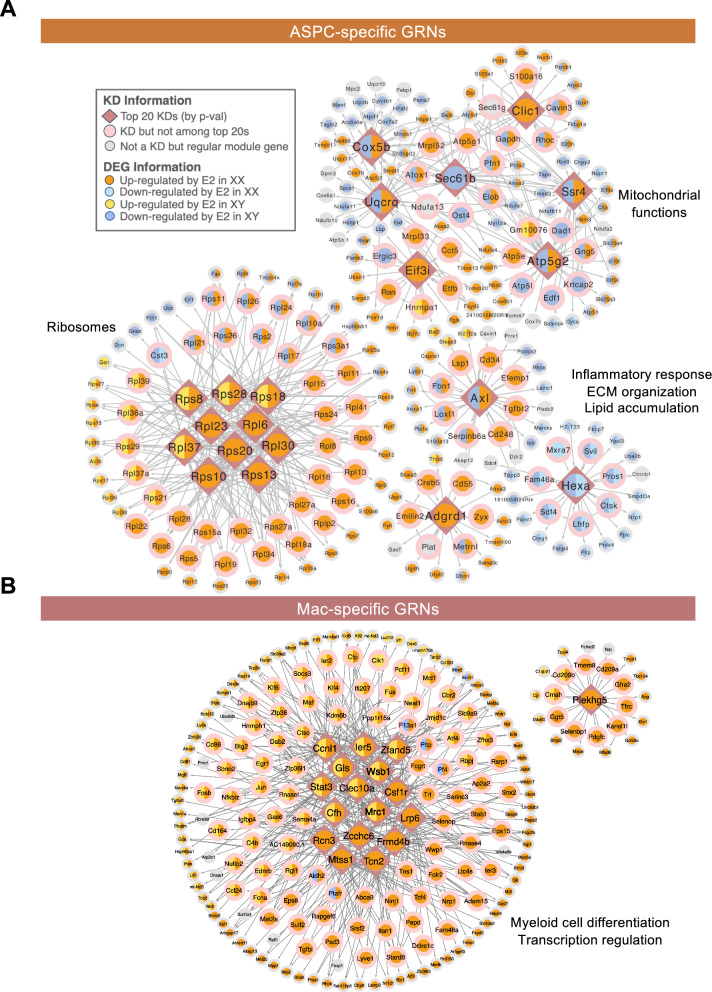
Cell-type-specific key driver (KD) analysis of E2-associated DEGs in XX and XY mice. (**A**) ASPC gene regulatory networks (GRNs) showing the top KDs. (**B**) Macrophage GRNs showing the top KDs. E2-associated DEGs from XX and XY mice were overlaid onto the cell-type-specific GRNs constructed from single-cell data with SCING. The top KDs, ranked by p-value, with their directly connected nodes, were selected to display. Enriched biological processes associated with the subnetworks are indicated alongside each network

The subnetworks of the top 20 KDs in ASPC (Fig. 4A) were enriched for molecular programs related to ribosome, progenitor/stem cell regulation, mitochondrial function, lipid metabolism, and immune regulation. Ribosomal genes encoding large or small ribosomal subunits comprised half of the top KDs, with four upregulated by E2 treatment in both sexes and six uniquely upregulated in XX mice. Mitochondrial function-associated KDs (*Atp5g2*, *Uqcrq*, *Cox5b*, *Ssr4*) encode components of oxidative phosphorylation complexes or related machinery, and were upregulated in XX but downregulated in XY mice, aligning with the opposing direction of mitochondrial function-related pathways enriched in ASPC between sexes (Fig. 2D). Immune related KD subnetworks centered around *Axl* and *Adgrd1* showed mostly upregulation by E2 in XX but downregulated in XY. The *Hexa*-centered module, linked to lipid metabolism-related processes, contained multiple KDs downregulated by E2 in both sexes.

Similarly, we constructed macrophage-specific GRNs to identify KDs whose neighboring genes were significantly enriched with E2-responsive macrophage DEGs. Among the top KDs (Fig. 4B), nine were concordantly upregulated by E2 treatment in both sexes, whereas eight were uniquely upregulated in XX mice. The subnetworks associated with these KDs were enriched for genes involved in myeloid cell differentiation (*Cd81*, *Maf*, *Mafb*, *Csf1r*, *Trf*, *Zfp36l1*, *Ninj1*, *Jun*, *Dab2*), anti-inflammatory macrophage markers (*Mrc1*, *Clec10a*, *Cd163*), and transcription regulation (*Stat3*, *Jun*, *Atf3*, *Klf4*, *Klf6*, *Zfp36*, *Runx1*, etc.).

These top ASPC and macrophage subnetworks revealed both sex-specific and shared regulatory networks of E2 treatment on adipose tissue remodeling.

### Sex-specific remodeling of ASPC-centric cell–cell communication in gWAT by E2

**Fig. 5:**
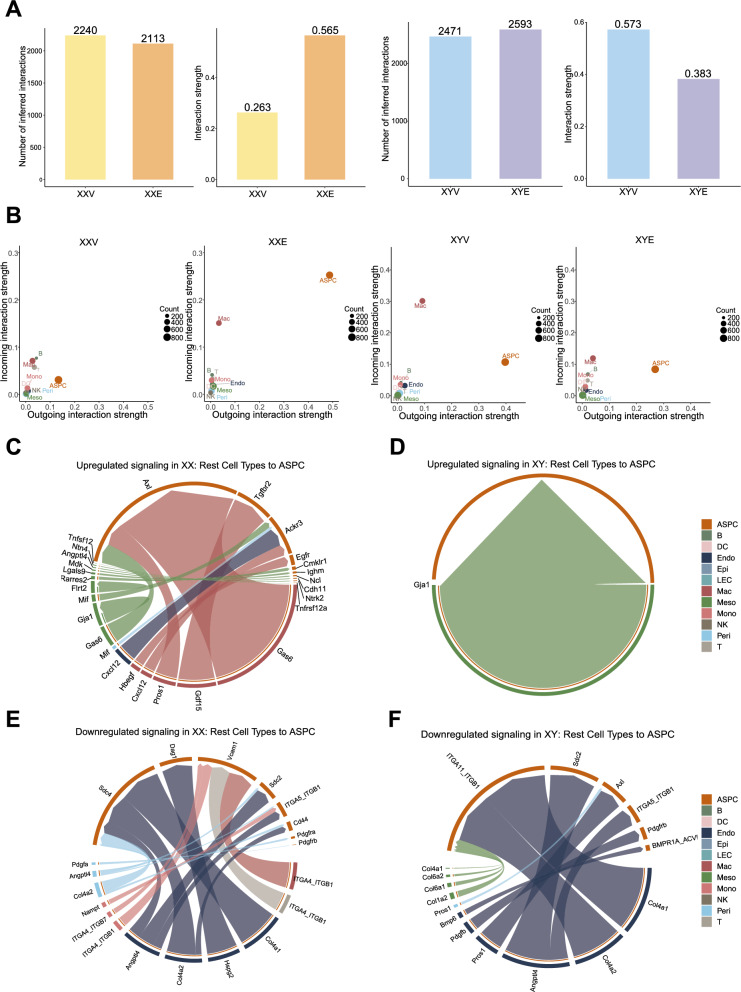
Cell–cell communication analysis in response to E2 treatment. (**A**) Number and strength of inferred cell–cell interactions in XX and XY mice with or without E2 treatment. (**B**) Strength of incoming and outgoing interaction in XX and XY mice with or without E2 treatment. **(C-D**) E2-induced upregulated signaling from other cell types to ASPCs in XX (**C**) and XY (**D**) mice. (**E–F**) E2-induced downregulated signaling from other cell types to ASPCs in XX (**E**) and XY (**F**) mice

To understand the potential role of E2 in remodeling cell–cell communications in the adipose microenvironment, we utilized CellChat [42] to conduct intercellular communication analyses, focusing on the interaction between ASPCs and other cell types. Interestingly, opposite patterns of interactions were observed between XX and XY mice: in XX mice E2 induced fewer intercellular interactions (2240 to 2113) but enhanced interaction strengths (0.263 to 0.565), whereas in XY mice E2 increased the number of interactions (2471 to 2593) but dampened interaction strength (0.573 to 0.383) in XY mice (Fig. 5A). When examining incoming and outgoing interactions, similar opposing patterns were observed between sexes for ASPC and macrophages (Fig. 5B). These opposing patterns suggest that E2 reshapes the intercellular signaling landscape in a sex-specific manner by tightening communication in females but diffusing it in males.

We further examined the ligand-receptor (LR) signaling between cell types with a focus on ASPC being the target cell type. In XX mice, the major cell types with upregulated signaling to ASPC were macrophages, mesothelial cells, endothelial cells, and pericytes, with macrophages exhibiting the strongest interaction strength across multiple LR pairs (Fig. 5C). Notable upregulated LR pairs included *Gdf15-Tgfbr2* and *Gas6-Axl* between macrophages and ASPC, agreeing with the roles of *Gdf15* and *Gas6-Axl* in the regulation of progenitor cell differentiation [60–62]. In XY mice, only one upregulated signaling pathway involving *Gja1-Gja1* gap junction from mesothelial cell to ASPC was identified (Fig. 5D). These findings indicate a female-biased pattern of E2-induced enhancement of macrophage to ASPC communication.

For downregulated LR signaling, in XX mice, endothelial cells, macrophages, monocytes, pericytes, and T cells showed weakened communication to ASPC, with endothelial cells displaying the greatest change (Fig. 5E) involving *Col4a1-Sdc4*, *Col4a2-Sdc4*, and *Col4a2-Cd44*. In XY mice, most downregulated LR pairs also originated from endothelial cell-ASPC interactions (Fig. 5F) involving *Angptl4-Sdc2*, *Angptl4-ITGA5_ITGB1*, *Col4a2-ITGA11_ITGB1*, and *Col4a1-ITGA11_ITGB1*.

We also explored the LR signaling from ASPC to other cell types altered in response to E2 (Fig. S7). Among the altered signaling, *Csf1-Csf1r* LR pair from ASPCs to macrophages was upregulated in XX mice, indicating signals from ASPC enhanced macrophage differentiation, survival and niche maintenance, agreeing with our observation on XX-specific, potentially beneficial remodeling of macrophage from the DEG and pathway analyses (Fig. S7A). In contrast, ASPC to macrophage upregulated signaling was relatively limited in XY mice (Fig. S7C). Among downregulated signaling from ASPC to macrophages, XX and XY mice exhibited some shared LR pairs, such as *Apoe/App-TREM2_TYROBP*, which is associated with lipid-sensing as well as activation pathways characteristic of *Trem2* + LAM (Fig. S7B, Fig. S7D). This also agrees with a reduced proportion of LAM in both sexes, suggesting a potential role of ASPC in mediating E2’s regulation of LAM activation through ligands such as APOE and APP.

### Sex- and cell-type-specific enrichment of E2-responsive genes with human metabolic traits

**Fig. 6:**
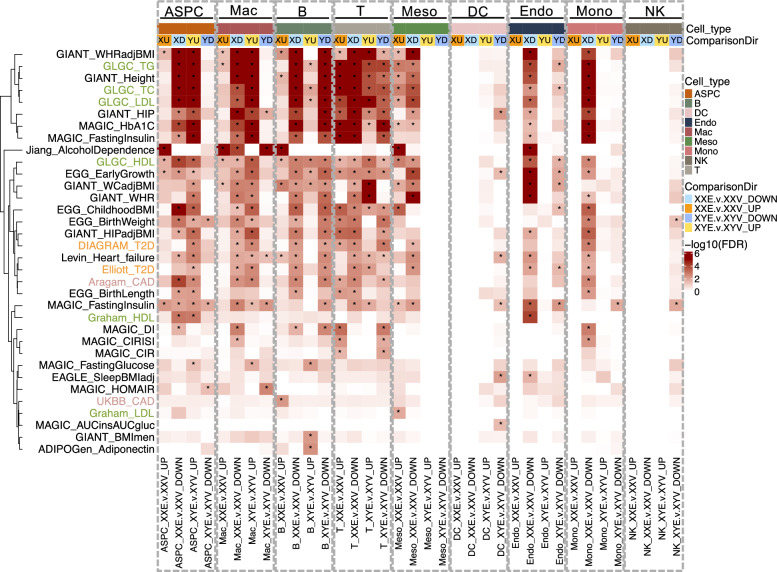
Enrichment of cell-type-specific E2-associated DEGs in XX and XY mice for human metabolic traits and disease-associated genes from GWAS studies. Enrichment analysis was performed using Marker Dependency Filtering (MDF) followed by Marker Set Enrichment Analysis (MSEA) in the Mergeomics pipeline. *, FDR < 0.05

Lastly, to examine the relevance of E2-affected genes identified in our mouse studies with human metabolic traits and diseases, we applied Mergeomics [40] MSEA to determine if the E2-induced genes in various cell types were significantly overrepresented with genes previously found to have SNPs associated with human metabolic traits and diseases from GWAS. We found that E2-induced DEGs showed cell-type- and sex-specific enrichment with human metabolic traits and diseases (Fig. 6). For ASPC and macrophage DEGs, E2-downregulated genes in XX and upregulated genes in XY mice were significantly enriched for GWAS hits associated with triglycerides (TG), total cholesterol (TC), low-density lipoprotein (LDL) cholesterol, heart failure, coronary artery disease, etc.. Interestingly, only upregulated ASPC DEGs in XY mice were enriched for T2D. For B cell DEGs, downregulated genes in both sexes showed concordant enrichment for GWAS signals of lipid profiles, glucose metabolism, BMI, T2D, and heart diseases; E2-induced upregulated DEGs in the XY, but not the XX, group were significantly enriched for SNPs associated with adiponectin levels and BMI of men. For endothelial, monocyte, and mesothelial cells, only DEGs from XX mice were enriched for GWAS signals of lipid profiles, BMI, glucose metabolism, and T2D. Across cell types, upregulated DEGs in XY but downregulated DEGs in XX tended to show association with cardiometabolic diseases/traits. These results suggest potentially opposite effects of E2 treatment in modulating cardiometabolic disease susceptibility in the two sexes.

## Discussion

In this study, we investigated the molecular and cellular effects of E2 treatment on gWAT in gonadectomized, diet-induced obese female and male mice through single-cell transcriptomics and network analyses. While both sexes exhibited reduced weight and adiposity under E2 treatment, XX mice demonstrated a more pronounced physiological and transcriptional response, suggesting a heightened sensitivity of female adipose tissue to E2. This result agrees with a recent study by Hope et al. [63], where the authors found that overexpression of adipose ERα had both sex-shared and specific effects on mice’s body composition (e.g., reduced total fat mass in both sexes with a more pronounced effect in female mice) as well as on certain gene expression levels of adipose tissue. Consistent with the stronger phenotypic effect of E2 in XX, stronger influences of E2 on adipose tissue composition and transcriptional programs were observed in females. We found that ASPCs and macrophages emerged as the most responsive populations to E2 treatment, likely with both direct effects of E2 through ER signaling and indirect effects through cell–cell crosstalk. In ASPCs, E2 elicited XX-specific transcriptional shifts across ASC and preA subtypes, and results from DEG, pathway, and network analyses converged on sex-biased regulation of stemness, translation, mitochondrial function, ECM remodeling, and adipogenesis. These coordinated findings suggest that E2 fine-tunes both metabolic and structural programs in female adipose progenitors. In macrophages, E2 consistently modulated inflammatory and polarization pathways in both sexes; however, integrative analyses revealed additional female-specific transcriptional regulation, indicating a higher degree of fine-tuning of immune responses in XX mice. While ER signaling was confirmed to be a central regulatory mechanism in E2 treatment effects based on TF network analysis across cell types and between sexes, many other transcription factors and regulators are involved in the cell-type and sex-specific regulation of E2-responsive genes. Many of the discordant E2-responsive genes between XX and XY showed significant enrichment for genes implicated in cardiometabolic diseases or traits in human GWAS.

Across cell types, E2 treatment had a particularly strong remodeling effect on ASPCs in XX mice, with an increase in ASPC proportion and distinct transcriptional shifts. In the ASPC subtype analyses, we observed a trend in increased ASC and decreased preadipocyte population fraction, especially in XX mice. Previous studies [64–66] indicated a potentially limited pool of multipotent stem cells in visceral WAT depots compared to subcutaneous WAT in mice. Our results suggest a XX-biased role of E2 in inducing ASPCs into a stem-like state while suppressing adipocyte differentiation in visceral gWAT. Agreeing with cell proportion results, E2 treatment also downregulated adipocyte differentiation genes *Spry1* [67–69] and *Lifr* [70] and upregulated stemness-associated *Ngfr* and *Cd34* [71], and translational regulators (*Rpl13*, *Eef1a1*, *Rack1*, and *Rps12*) in XX mice, further supporting that E2 may promote a progenitor-like state while limiting adipogenic commitment in XX mice. Our recent work [70] identified *Lifr* as a key adipogenesis activator, found to be “unlocked” specifically in visceral adipose tissue of early-aged male mice. The current study provides novel insight into the potential suppressive role of E2 treatment on *Lifr* gene in XX mice, which may contribute to adipogenesis inhibition and lowered adiposity in female mice. Additionally, in both sexes, E2 treatment suppresses genes in ASPCs related to ECM remodeling and deposition (*Postn*, *Bgn*, *Sparc*), with XY mice showing stronger downregulation of additional ECM- and fibrosis-associated genes, including *Col1a2*, *Aspn*, and *Mmp2*, particularly in preadipocytes. This pattern points to a more pronounced modulation of ECM turnover in XY mice. Notably, prior studies have reported that these ECM-related genes are upregulated in obesity, contributing to fibrotic and dysregulated remodeling of adipose tissue [72, 73]. Thus, their downregulation by E2 treatment, especially in XY mice, may indicate a male-biased role of E2 in alleviating fibrosis and excessive ECM deposition. Pathway analyses reinforced these patterns, with adipogenesis and lipid metabolism pathways selectively suppressed and ribosomal pathways upregulated in XX ASPCs and ER stress responses and collagen metabolism reduced in both sexes. The opposite regulation of cellular respiration and ATP metabolism genes (up in XX, down in XY) further points to divergent energy states regulated by E2 between sexes in ASPCs.

Further supporting the cell proportion, gene, and pathway findings above, our TF enrichment analysis of ASPC DEGs affected by E2 revealed XX-specific enrichment of TEAD4 and TCF4, which are involved in stem cell maintenance and fate determination [74, 75], as well as mesenchymal lineage determination and adipogenesis regulation-related RUNX [76, 77], suggesting sex-specific differences in regulatory pathways involved in cell differentiation in ASPC. This result aligns with a previous study, which showed that adipogenesis inhibition by ER cellular pools was partially mediated by AKT signaling and Wnt-β-catenin-TCF4 pathway to inhibit PPARγ expression and progenitor differentiation to mature adipocytes in 3T3-L1 preadipocytes and adipose-derived stem cells isolated from visceral adipose fat depot in ovary-intact female mice [78]. In addition to identifying transcription factors, our GRN analysis highlighted upregulated ribosomal and mitochondrial genes as additional central hubs of E2-responsive regulation in ASPCs, particularly in XX mice. This pattern suggests that E2 may selectively enhance overall biosynthetic and energy-producing capacity in female ASPCs, potentially supporting the maintenance of progenitor characteristics. Consistently, Zhou et al. [79] reported that mitochondrial, transcriptional, and protein transport processes were markedly altered following adipose tissue-specific ERα deletion in female FERKO mice, which also exhibited increased adipose tissue mass. Their findings also further indicated that ERα signaling modulates the kinetics of protein turnover, highlighting its broader role in coordinating metabolic and proteostatic programs in adipose tissue of female mice. In contrast, mitochondrial modules displayed opposite regulation by E2 between sexes: OXPHOS-related regulators (*Atp5g2*, *Uqcrq*, *Cox5b*) were upregulated in XX but downregulated in XY mice, which may drive distinct metabolic states and consequences for proliferation and differentiation potential. Consistent with our findings, a recent study [80] in human fibroblasts reported sex-specific opposite effects of estradiol, where E2 upregulated genes involved in bioenergetics and proteostasis in the female fibroblasts but downregulated these genes in male fibroblasts.

Our intercellular communication analysis also revealed potential cell crosstalk mechanisms that drive sex-biased ASPC reprogramming by E2. For instance, LR pair *Gdf15-Tgfbr2* between macrophage and ASPC was upregulated in XX but not in XY mice. *Gdf15* (growth factor 15, also known as macrophage inhibitory cytokine 1) is a stress-responsive cytokine of the transforming growth factor-beta (TGF-beta) superfamily. Previous studies suggested that macrophages can secrete GDF15, which has been suggested to modulate energy homeostasis by improving insulin resistance and promoting lipolysis in adipose tissue [60]. GDF15 has also been reported to have a role in inhibiting early-stage adipocyte differentiation in pre-adipocytes. *Gas6-Axl* between macrophage and ASPC was another XX-specific LR pair upregulated by E2. *Gas6* (Growth arrest-specific gene 6) is expressed, produced, and released by macrophages [81], adipocytes, endothelial cells, and other cell types [82]. *Gas6* expression was shown to be lower in the gWAT of obese ob/ob mice compared to wild-type mice, and decreased as human preadipocytes differentiated into adipocytes in vitro [83]. In mouse models, a transient increase in *Gas6* expression in proliferating preadipocytes was associated with growth arrest that maintains preadipocytes in an undifferentiated state, whereas its expression decreased as soon as adipogenic medium was added [84]. *Axl*, a gene that encodes a member of the receptor tyrosine kinases TAM (*Tyro3*, *Axl*, and *Mer*) and the major receptor for *Gas6* [85], is restricted to the adipose stromal vascular fractions, including pre-adipocytes. AXL is involved in promoting cellular growth and survival in cancer cells upon exogenous GAS6 stimulation [86]. While *Axl* deficiency was shown not to contribute to adipogenesis in vitro or in vivo [87], whether increased *Axl* signaling through *Gas6* stimulation modulates ASPC proliferation and differentiation remains uncertain. In addition, E2 dampens ECM-related LR pairs mainly from endothelial cells to ASPC in both XX and XY mice, supporting our DEG and pathway results. Therefore, the stemness in ASPCs in XX mice appears to be controlled by macrophages, whereas the ECM remodeling in both sexes is likely controlled by endothelial-ASPC signaling.

In macrophages, E2 treatment showed modestly reduced obesity-related LAM subtype but increased PVM in the gWAT of both sexes, agreeing with the previous mouse epididymal WAT study by Sarvari et al. [25], where lean mice had a lower percentage of LAM and a higher percentage of PVM compared to obese mice. Consistent with the cell fraction changes, E2 broadly suppressed LAM markers *Trem2* and *Cd9* [88] and concordantly downregulated pro-inflammatory mediators *Lgals3*, *Fabp5*, and *S100a4* as well as mitochondrial function genes in both sexes. XY-specific suppression of *Lgals1*, *Ccl6*, *Ftl1*, and *Fth1* suggested further attenuation of inflammatory and metabolic programs in XY mice, while opposite regulation (downregulated in XX; upregulated in XY) of mitochondrial and antigen presentation genes (*Lars2*, *H2-Ab1*, *H2-Aa*, *H2-Eb1*, *H2-Q7*) indicated sex-dependent immune and metabolic modulation. XX-specific downregulation of phagocytosis, lipid storage, and antigen presentation, coupled with unique upregulation of wound healing and cell growth, further highlighted targeted and likely beneficial remodeling of macrophage functions in XX mice.

Our macrophage network analysis suggests that E2 modulates regulatory programs governing macrophage differentiation and transcriptional control, with a shared core response across sexes and additional sex-specific regulation in XX mice. The concordant upregulation of network key drivers and module genes such as *Maf*, *Mafb,* and *Stat3* is consistent with prior studies showing that these transcription factors are central to macrophage polarization and anti-inflammatory activity [89, 90]. The enrichment of anti-inflammatory markers (*Mrc1*, *Clec10a*, *Cd163*) further supports a role for E2 in promoting a reparative, M2-like phenotype, which aligns with earlier reports that estrogen dampens pro-inflammatory macrophage activation and enhances tissue-protective responses in adipose and vascular contexts [63, 91, 92]. The observation that XX mice exhibited an additional layer of regulatory changes suggests that E2 may elicit more nuanced transcriptional control in female-derived macrophages, a concept that has been proposed in prior studies highlighting sex differences in estrogen receptor signaling and macrophage plasticity in alveolar macrophages in lungs of mice [93]. This female-biased E2 modulation of macrophages further explains the unique macrophage-ASPC communication to control ASPC stemness in XX mice that we uncovered through the cell crosstalk analysis. Moreover, in addition to the direct effects of E2 on macrophages via ER signaling, as illustrated through the sex-concordant estrogen receptor enrichment in the TF analysis, ASPC-macrophage cell–cell signaling results also suggest sex-shared and unique indirect effects of E2 on macrophage remodeling, partially mediated through ligand-receptor signaling from ASPCs. Together, these findings imply that E2 not only broadly reprograms macrophage transcriptional networks but also introduces sex-dependent refinements, which could underlie differential susceptibility to metabolic or inflammatory outcomes between sexes.

Lastly, by integrating E2-induced DEGs with human metabolic GWAS data, we found striking sex- and cell-type-specific enrichment patterns. In ASPCs and macrophages, XX downregulated and XY upregulated DEGs were enriched for cardiovascular and lipid traits, suggesting opposing roles of E2 in disease susceptibility between sexes. This dichotomy was not observed in B cells, where DEGs from both sexes showed consistent enrichment. Interestingly, enrichment of disease-associated genes among DEGs in endothelial, mesothelial, and monocyte populations was found only in XX mice, supporting the notion that female-specific transcriptional responses in these cell types may further modulate differential susceptibility to metabolic disorders such as T2D, dyslipidemia, and cardiovascular disease.

We acknowledge the following limitations of the study. First, our estradiol measurement was performed with an ELISA kit, which has limited sensitivity in the sub-physiological range typical of gonadectomized mice, which can artificially inflate variability and add difficulty in interpreting absolute values in the vehicle group. For this reason, the interpretation should focus on the increases in the E2 treatment group compared to the vehicle group, and the dose-dependent effect should be pursued in the future. We note that a more precise quantification of circulating estradiol, using high-sensitivity LC–MS/MS, would validate hormonal changes in the model. Alternatively, uterine weight can be recorded as an additional indicator of estrogenic activity in female mice. Second, the interpretation of the results should consider the age at which the hormone was administered. E2 treatment began at 33 weeks of age, corresponding to the early aging phase in mice. As a result, the observed responses may reflect age-specific interactions between E2 and the aging adipose tissue environment, rather than generalizable effects across the lifespan. Future studies administering E2 at different ages or across developmental windows will be important to disentangle age-dependent sensitivity from E2-specific effects. Third, our transcription factor enrichment relied on databases containing ChIP-seq and ChIP-ChIP data from various tissues of mouse models, which lacked visceral adipose-specific estrogen receptor ChIP-seq data. Future studies to directly map ER binding in visceral adipose cell types are warranted. Fourth, due to limited access to laboratory resources during the COVID pandemic, we did not conduct immunohistochemical staining for macrophages in gWAT nor perform flow-cytometry-based profiling to assess inflammatory cell populations after estradiol supplementation in our study. Therefore, subsequent targeted analyses, such as CD68 or F4/80 immunostaining, or multiparameter flow cytometry, are warranted to directly quantify macrophage infiltration and phenotype changes associated with estradiol treatment. We also did not measure adipocyte size in the current study, although in our recent independent study [94], we showed that estrogen reduced adipocyte size in males, thereby reducing hypertrophy. Lastly, functional validations at cell-type-specific gene (e.g., qPCR on altered ASPC and macrophage DEGs), protein, and network levels will also be required to confirm the inferred effects of E2 on transcriptional programs in both sexes.

### Perspectives and Significance

Our work reveals how E2 reprograms visceral adipose tissue at single-cell resolution, highlighting ASPCs and macrophages as the most responsive cell types. While some regulatory programs such as ER signaling are conserved, others diverge between sexes, suggesting that E2’s actions on the adipose tissue are shaped by sex chromosome complement. These insights provide a mechanistic basis for understanding sex differences in adipose biology and disease susceptibility, which may inform future sex-specific therapeutic approaches to obesity and metabolic disease.

## Conclusions

Our study demonstrates that estrogen exerts sex- and cell-type dependent effects in visceral adipose tissue in obese mice. Females exhibited stronger effects on body weight and fat reduction as well as transcriptional remodeling in ASPCs and macrophages, reflecting enhanced sensitivity of female adipose tissue to estrogen. Our findings reveal that estrogen regulates not only metabolic and structural programs but also intercellular communication in a sex-specific manner. By linking estrogen-responsive genes to human metabolic traits, our work provides mechanistic insight into why women and men differ in their susceptibility to metabolic diseases. Understanding these sex-dependent regulatory pathways may inform the development of personalized, hormone-based strategies for treating metabolic disorders.

## Supplementary Information

Additional file 1.

Additional file 2.

Additional file 3.

## Data Availability

The datasets generated and/or analyzed during the current study are available in the Single Cell Portal and can be accessed with study number SCP3381.
