# Supplementary material for: Estradiol treatment induces both shared and unique gene regulation and networks in adipose cell types of gonadectomized obese XX and XY mice
Source: Biol Sex Differ. 2026 Feb 23;17:41. doi: 10.1186/s13293-026-00859-z (PMC12930959; doi:10.1186/s13293-026-00859-z)
Supplement: Supplementary file 1 — Additional file 1. [file 13293_2026_859_MOESM1_ESM.docx]

Supplementary Materials for

**Estradiol Treatment Induces Both Shared and Unique Gene Regulation and Networks in Adipose Cell Types of Gonadectomized Obese XX and XY Mice**

Yutian Zhao, *et al.*

Corresponding authors:

John M. Stafford ([John.Stafford@osumc.edu](mailto:John.Stafford@osumc.edu))

Xia Yang ([xyang123@ucla.edu](mailto:xyang123@ucla.edu))

### Supplementary figures

**Figure S1**. **Phenotypic measurements and single-cell atlas of gonadal adipose SVF in diet-induced obese, gonadectomized mice.** (**A**) Circulating E2 levels across four genotypes measured at Week 47 from EDTA-anticoagulated plasma (n = 6-8/sex/treatment). (**B**) Longitudinal body weight measurements across four genotypes from Week 31 to Week 47. (**C**) Percentage of fat mass across four genotypes at Week 30 and at Week 40 (n = 6-8/sex/treatment). (**D-F**) Circulating coagulation factors 7, 8, and 10 across four genotypes measured at Week 47 (n = 6-8/sex/treatment). * *p* < 0.05, ** *p* < 0.01, *** *p* < 0.001, **** *p* < 0.0001 in XX mice (XXE vs XXV); ^#^ *p* < 0.05, ^###^ *p* < 0.001 in XY mice (XYE vs XYV); ^$^, *p* < 0.05 between XXE vs XYE mice.

**Figure S2. Overview of scRNA-seq quality control, annotation, and cell type composition.** (**A**) Violin plots showing the number of UMIs, number of detected features, and percentage of mitochondrial reads per cell across samples after filtering low-quality cells. (**B**) Comparison of top 200 marker genes from annotated cell types with the top 200 marker genes from reference cell types reported in the mouse adipose study by Emont et al. [1]. (**C**) Uniform Manifold Approximation and Projection (UMAP) visualization of all cells colored by sample ID. (**D**) Euclidean distance based on top 2,000 highly variable genes comparing E2-treated vs vehicle groups in XX and XY mice across cell types. (**E**) Proportional distribution of cell types across samples.

**Figure S3. Subtype analysis of Adipose Stem and Progenitor Cells (ASPCs).** (**A**) UMAP visualization showing annotated ASPC subtypes. ASC, adipose-derived stem cell; preA, preadipocyte; Areg, adipogenesis regulator. (**B**) Expression patterns of canonical marker genes across ASPC subtypes. (**C**) UMAP visualization of ASPC cells colored by genotype. (**D**) UMAP visualizations of ASPC subtypes separated by genotype. (**E**) Proportional representation of ASPC subtypes using two-way ANOVA followed by Tukey’s HSD post-hoc test.

**Figure S4. Subtype analysis of macrophages.** (**A**) UMAP visualization showing annotated macrophage subtypes. LAM, lipid-associated macrophage; P-LAM, proliferating LAM; PVM, perivascular-like macrophage; NPVM, non-perivascular-like macrophage; CEM, collagen-expressing macrophage. (**B**) Expression patterns of canonical marker genes across macrophage subtypes. (**C**) UMAP visualization of macrophage cells colored by genotype. (**D**) UMAP visualizations of macrophage subtypes separated by genotype. (**E**) Proportional representation of macrophage subtypes using two-way ANOVA followed by Tukey’s HSD post-hoc test.

**Figure S5. Stratified rank-rank hypergeometric overlap (RRHO) analysis.** (**A-J**) Stratified RRHO results between XXE vs. XXV (x-axis) and XYE vs. XYV (y-axis) across all cell types in adipose SVF tissue. (**A**) ASPCs. (**B**) Macrophages. (**C**) Mesothelial cells. (**D**) B cells. (**E**) T cells. (**F**) Monocytes. (**G**) Endothelial cells. (**H**) Natural killer cells. (**I**) Pericytes. (**J**) Dendritic cells. (**K**) Illustration of interpretation of stratified RRHO2 results [2].

**
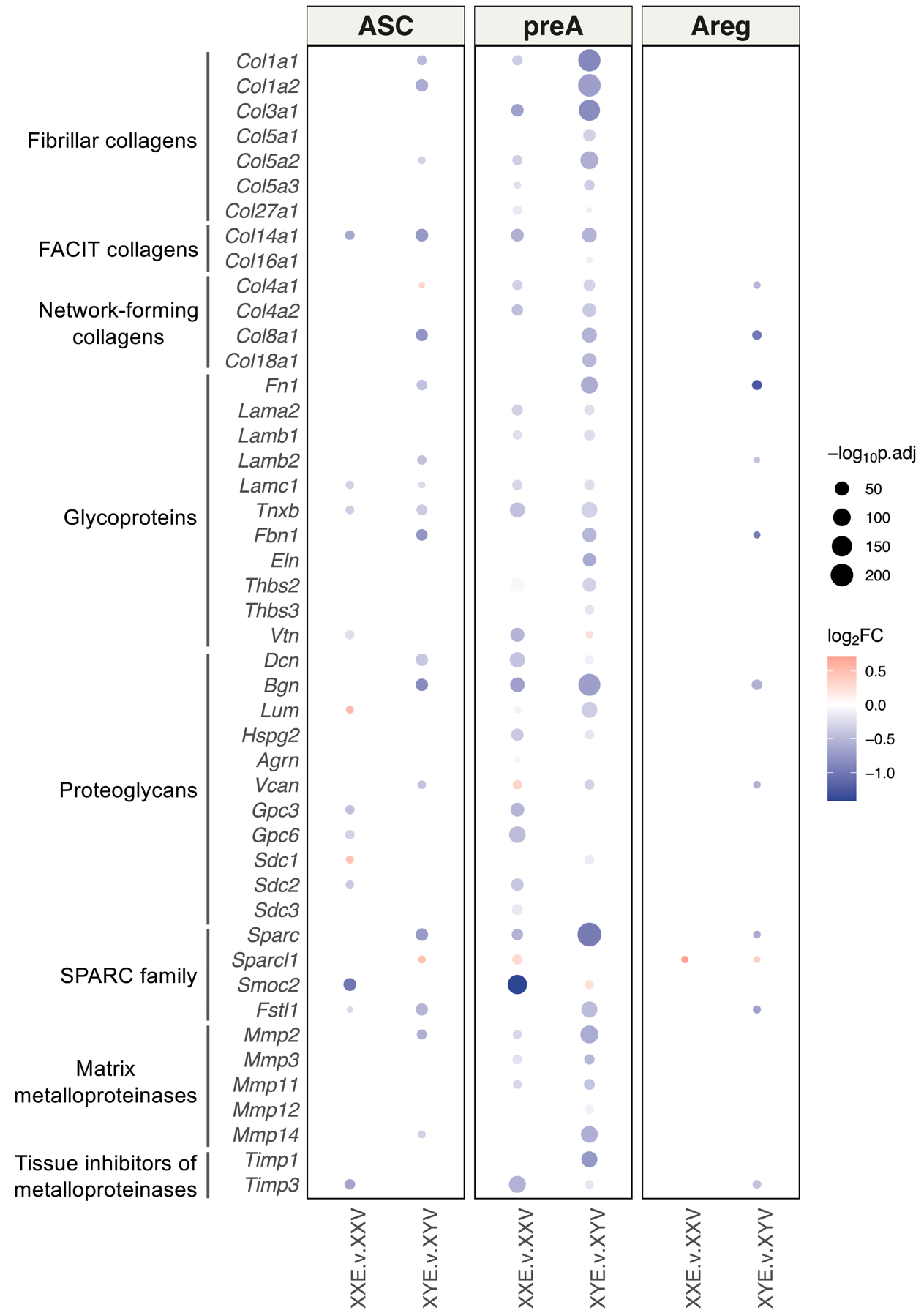
Figure S6. Dot plot showing differential expression of genes encoding proteins and enzymes involved in ECM synthesis and degradation across ASPC subtypes in XX and XY mice.** FACIT, Fibril-Associated Collagens. SPARC, Secreted Protein, Acidic and Rich in Cysteine.

**Figure S7. Cell-cell communication analysis from ASPC to the other cell types in response to E2 treatment.** (**A-B**) E2-induced up- (**A**) and down-regulated (**B**) signaling from ASPC to rest cell types in XX mice. (**C-D**) E2-induced up- (**C**) and down-regulated (**D**) signaling from ASPC to the other cell types in XY mice.

### Supplementary tables

**Table S1. Cell-type-specific differentially expressed genes between E2 and vehicle groups in XX and XY mice.**

**Table S2. Cell-type- and sex-specific pathway enrichment results.**

**References**

1. Emont MP, Jacobs C, Essene AL, Pant D, Tenen D, Colleluori G, et al. A single-cell atlas of human and mouse white adipose tissue. Nature. 2022;603:7903:926–33; doi:10.1038/s41586-022-04518-2.

2. Cahill KM, Huo Z, Tseng GC, Logan RW, Seney ML, Cahill KM, et al. Improved identification of concordant and discordant gene expression signatures using an updated rank-rank hypergeometric overlap approach. Scientific Reports 2018 8:1. 2018–06–25;8:1; doi:10.1038/s41598-018-27903-2.
